# Research progress on intervention strategies for post‐traumatic stress in military personnel exposed to cold environments and radiological threats

**DOI:** 10.1002/gps3.70061

**Published:** 2026-10-29

**Authors:** Hui Wang, Yongxian Zhang, Chuanyu Zhu, Dawei Zhang, Peng Zhou, Chenglin Sang

**Affiliations:** ^1^ Department of Orthopedics the 960th Hospital of the Joint Logistic Support Force of the Chinese People's Liberation Army Jinan Shandong China; ^2^ Department of Thyroid and Breast Surgery the 960th Hospital of the Joint Logistic Support Force of the Chinese People's Liberation Army Jinan Shandong China

## INTRODUCTION

In China, cold regions encompass much of Northeast, Northwest and North China and account for 44.5% of the country's total land area.^1^ Prolonged deployment in these environments exposes service members to multiple physiological and psychological stressors, including extreme cold, extended winter seasons, elevated ultraviolet radiation, topographically complex terrain, logistical constraints, social isolation, limited access to information, strict military discipline and intensive combat‐readiness training. Collectively, these factors may increase vulnerability to post‐traumatic stress disorder (PTSD) and associated adverse psychological outcomes, including anxiety, depression, emotional numbing and hypervigilance.^2^

Although nuclear and radiological accidents share features with other anthropogenic disasters, they also present distinctive challenges. Central to such incidents is exposure to ionising radiation, a physical hazard that cannot be perceived by the senses. Nuclear and radiological events are also strongly associated in the public consciousness with nuclear weapons and explosions, which may amplify psychological apprehension. Military personnel may therefore experience pronounced psychological distress in response to these incidents.^3^ Prolonged deployment in environments involving a radiological threat may further increase the risk of PTSD. The current global nuclear security landscape—marked by heightened geopolitical tensions, including the continuing Russia–Ukraine conflict—has generated tangible consequences for nuclear safety and security. Notably, operational instability at some nuclear facilities has exposed troops in potential conflict zones to sustained anxiety regarding radiation exposure.^4^, ^5^ The combination of chronic psychological stress and acute combat‐related hazards may further elevate the risk of PTSD. However, research on PTSD interventions in cold‐region military settings involving radiological threats remains limited. This paper summarises the mechanisms that may contribute to PTSD in this context and examines potential intervention strategies.

### Pathogenesis of PTSD and the impact of environmental factors

PTSD is a chronic mental disorder that can develop after exposure to a traumatic event. Its clinical features include intrusive symptoms, avoidance, negative alterations in cognition and mood and heightened arousal. It is often accompanied by secondary negative emotions such as depression and anxiety.^6^, ^7^

### Physiological mechanisms

Combined exposure to cold and ionising radiation exerts profound pathophysiological effects across multiple organ systems, particularly the nervous, haematopoietic, cardiovascular and immune systems. Potential effects include neuronal apoptosis, synaptic remodelling, haematopoietic suppression, endothelial dysfunction and altered immune function. Cold and radiation exposure may also disrupt neuroendocrine regulation, including the hypothalamic–pituitary–adrenal (HPA) axis, central neural circuits and the brain–gut–microbiota axis. Both cold and radiation‐related stress may increase HPA‐axis activity and cortisol secretion. Prolonged exposure may also contribute to hippocampal atrophy and prefrontal cortical dysfunction. HPA‐axis dysregulation has been associated with adverse emotional states, such as depression and anxiety.^8^ Critically, these systemic pathophysiological perturbations compromise whole‐body homoeostasis via bidirectional cross talk within the neuroendocrine–immune network, thereby exerting direct, persistent and cumulative adverse effects on psychological resilience and mental health.^9^, ^10^ In cold‐region operational settings involving nuclear threat exposure, the synergistic interaction between hypothermic stress and radiobiological toxicity establishes a dual‐stress paradigm. This convergence amplifies multisystem dysregulation and neuroimmune–neuroendocrine disturbance. Such effects could provide a physiological and pathological substrate for the onset, persistence or exacerbation of PTSD symptoms in this high‐risk population.

Cold exposure is a well‐established contributor to sleep architecture disruption, including delayed sleep onset, reduced total sleep time, diminished sleep efficiency and impaired restorative function—changes that directly compromise affective regulation. Prolonged thermal stress among military personnel deployed in cold regions has also been associated with a higher prevalence of functional gastrointestinal disorders. The biological effects of ionising radiation are dose dependent: Haematopoietic suppression predominates at lower doses, whereas moderate doses can cause gastrointestinal mucosal injury and barrier dysfunction.^11^, ^12^ In military personnel exposed to multiple operational stressors, changes in the gut microbiota and its metabolites have been associated with intestinal permeability, systemic inflammation, gastrointestinal symptoms and psychological and emotional outcomes.^13^

Karl et al.^14^ characterised a 4‐day Arctic winter military training exercise involving extreme energy expenditure, an energy deficit, sustained cold exposure and severe sleep restriction as a model of the multiple stressors encountered during high‐readiness military training and combat deployment. They identified time‐dependent changes in the composition and function of the gut microbiota, including pronounced alterations in taxonomic diversity, the relative abundance of key bacterial genera and microbially derived metabolites. These changes coincided with increased intestinal permeability and higher concentrations of systemic inflammatory markers, including interleukin‐6 and C‐reactive protein.^15^ Modulation of the gut microbiota may therefore warrant investigation as a potential approach to the prevention or treatment of PTSD in personnel operating under extreme environmental conditions.

### Psychological mechanisms

Extreme cold can act as a substantial psychological stressor, contributing to mental fatigue and adverse emotional states among military personnel. Psychological difficulties may be particularly pronounced among new recruits, personnel with higher levels of education and those assigned to outdoor duties. The reported prevalence of depressive symptoms was 37.5%, and anxiety frequently co‐occurs with depression and may contribute to acute anxiety disorders. Cold‐related sleep disturbance may also impair attention, working memory and other cognitive functions.^16^ The reported prevalence of PTSD among soldiers stationed in high‐altitude cold regions was 7.8%, significantly higher than that in the general population. Soldiers in cold regions may also have lower psychological resilience than those stationed in warmer regions. Their capacity to adapt, withstand stress and recover may therefore be challenged by the combined effects of the environment and operational demands.^17^

Because ionising radiation is imperceptible and strongly associated with catastrophic nuclear hazards, nuclear and radiological accidents may elicit profound and sometimes disproportionate fear.^18^ The resultant psychosocial consequences can extend well beyond the effects predicted by the actual radiological risk. Misinformation and rumours may amplify these responses, escalating local distress into widespread community‐level anxiety or collective panic. Furthermore, some exposed individuals develop persistent psychological hyperarousal and heightened threat vigilance, increasing their risk of generalised anxiety disorder, major depressive disorder and PTSD even in the absence of direct physical injury.^19^ Monitoring of radiation‐related physical harm should therefore be accompanied by appropriate psychological support for affected personnel.

In summary, compared with either cold or radiological exposure alone, their combination presents three distinctive challenges. First, their physiological effects may be mutually reinforcing. Second, personnel must cope with both the physical discomfort of extreme cold and fear arising from the invisible nature of radiation and its association with nuclear weapons. Third, intervention delivery may be constrained by geographical isolation, limited resources and impaired operation of electronic devices at very low temperatures. These challenges support the development of a tiered intervention system tailored to this setting.

## INTERVENTION AND TREATMENT STRATEGIES

Established treatments for military‐related PTSD include pharmacotherapy and psychological interventions such as cognitive processing therapy (CPT), eye movement desensitisation and reprocessing and trauma‐focused therapies. These approaches are supported by a substantial body of evidence and form the basis of current clinical guidelines.^20^ Interventions for PTSD in personnel exposed to the combined stressors of cold and radiological threats remain an important research priority. We therefore organise existing intervention strategies into a three‐tier framework: Tier 1, universal resilience building; Tier 2, targeted screening and personalised treatment; and Tier 3, specialised rehabilitation.

### Tier 1—Universal resilience building

Military personnel routinely operate in harsh environments and face multiple concurrent stressors. Psychological resilience is central to coping with adversity and maintaining mental health and therefore represents a first line of defence. Resilience‐building measures should be implemented proactively across deployed forces through a structured, evidence‐based programme. The US Army's Comprehensive Soldier Fitness programme proposed by Martin Seligman provides a possible model. It comprises three components^21^: global assessment, self‐improvement and the training of master resilience trainers. First, participants undergo a multidimensional assessment tailored to the operational environment, including measures of physical health, such as body composition and blood glucose, and physical fitness, such as long‐distance running and sit‐ups, while taking account of the demands imposed by cold and potential radiation exposure. The Global Assessment Tool (GAT) assesses emotional, social, family and spiritual fitness and can help identify personnel experiencing psychological stress. All relevant personnel complete the assessment and undergo regular reassessments. Second, GAT results inform personalised self‐improvement plans and targeted training across the four domains. For personnel operating in cold and radiological threat environments, particular emphasis could be placed on emotional regulation, interpersonal communication and psychological preparedness. The third component is the preparation of master resilience trainers, who can be embedded in frontline units. Their training could be tailored to operational demands, including offensive operations and responses to surprise attacks, while covering core resilience skills such as self‐awareness, avoidance of thinking traps and identification of character strengths. Once trained, these instructors can deliver group‐based resilience training to frontline personnel.^22^

### Tier 2—Targeted screening and personalised treatment

For individuals who remain at elevated risk despite universal measures, Tier 2 involves targeted assessment to support early, personalised intervention. Personalised medicine holds considerable promise in the treatment of military‐related PTSD. Improving treatment selection for personnel exposed to cold environments and radiological threats will require systematic characterisation of the clinical and biological features of PTSD in this population. A multidimensional biopsychosocial assessment framework should integrate environmental stressors, potential radiation injury and psychological trauma.

Neuroimaging studies have identified functional abnormalities in brain regions involved in emotional processing among people with PTSD. These include abnormal activities in the amygdala, insula and dorsal anterior cingulate cortex during emotional response and salience processing, together with impaired regulatory function in the medial prefrontal cortex, lateral prefrontal cortex and ventral anterior cingulate cortex.^23^ Prolonged nuclear exposure in cold regions may further exacerbate functional imbalances in these brain regions through oxidative stress, sustained hyperactivity of the HPA axis and chronic neuroinflammation. Patterns of neural dysfunction may also vary according to radiation dose, deployment duration and cold tolerance, rendering accurate subtyping difficult using a single scale.^24^ Advances in functional magnetic resonance imaging (fMRI) and electroencephalography (EEG), combined with the detection of biomarkers and genetic data, may help characterise heterogeneity and identify potential intervention targets.

Large‐scale randomised controlled trials of first‐line interventions, including psychoeducation therapy, EEG alpha wave feature‐matched CPT and exposure therapy, have generated datasets that can be used for the training and validation of machine learning algorithms.^25^ These models may help predict which intervention is most likely to benefit an individual patient. This ‘big data‐driven’ research paradigm not only facilitates the optimisation and refinement of treatment plans and their adaptation to different clinical settings but also provides an objective and evidence‐based scientific basis for clinical decision‐making through continuous quality improvement. In summary, machine learning analysis of large datasets is expected to advance personalised treatment for PTSD by enabling patients in cold‐region radiological environments to be matched more precisely with the most appropriate intervention.

### Tier 3—Specialised rehabilitation supported by digital technologies

For personnel diagnosed with PTSD, Tier 3 offers specialised rehabilitation programmes tailored to the operational environment, using various novel technology‐assisted interventions as scalable, portable adjuncts to psychological treatment.

### Applications of virtual reality (VR)

VR technology uses computer‐generated environments and sensory devices to create immersive experiences. With its high‐fidelity reproduction capability of real‐world scenes, VR technology has increasingly been used in military medical research and practice worldwide.^26^ In settings involving extreme cold or a high risk of radiation exposure, VR technology may overcome the environmental limitations and safety barriers of field‐based research. The war in Ukraine has highlighted the need for accessible mental health interventions when anxiety and depression are prevalent among military personnel and conventional rehabilitation resources are limited. A randomised controlled trial evaluated the immediate changes in mood and anxiety following a professionally designed VR intervention incorporating evidence‐based psychological treatment techniques. Participants viewed 360° videos while seated using a Meta Quest 2 head‐mounted device, as shown in figure 1. The results of the Hospital Anxiety and Depression Scale assessment indicated that VR therapy could rapidly improve immediate mood and alleviate anxiety. After the intervention, the anxiety and depression symptoms of the participants decreased significantly by 14.5% and 12.3%, respectively, and the findings were consistent.^27^ The study suggested that immersive 360° video‐based VR therapy may provide an effective and accessible means of addressing the psychological effects of war, particularly where conventional mental health services are limited. VR can also simulate scenarios such as cold‐region field medical operations and emergency responses to radiation exposure, providing a safe and controlled environment for military medical research, practical training and rehabilitation in challenging operational settings. It may therefore represent a useful tool for the continued development of military medicine in cold environments involving potential radiation exposure.

**FIGURE 1:**
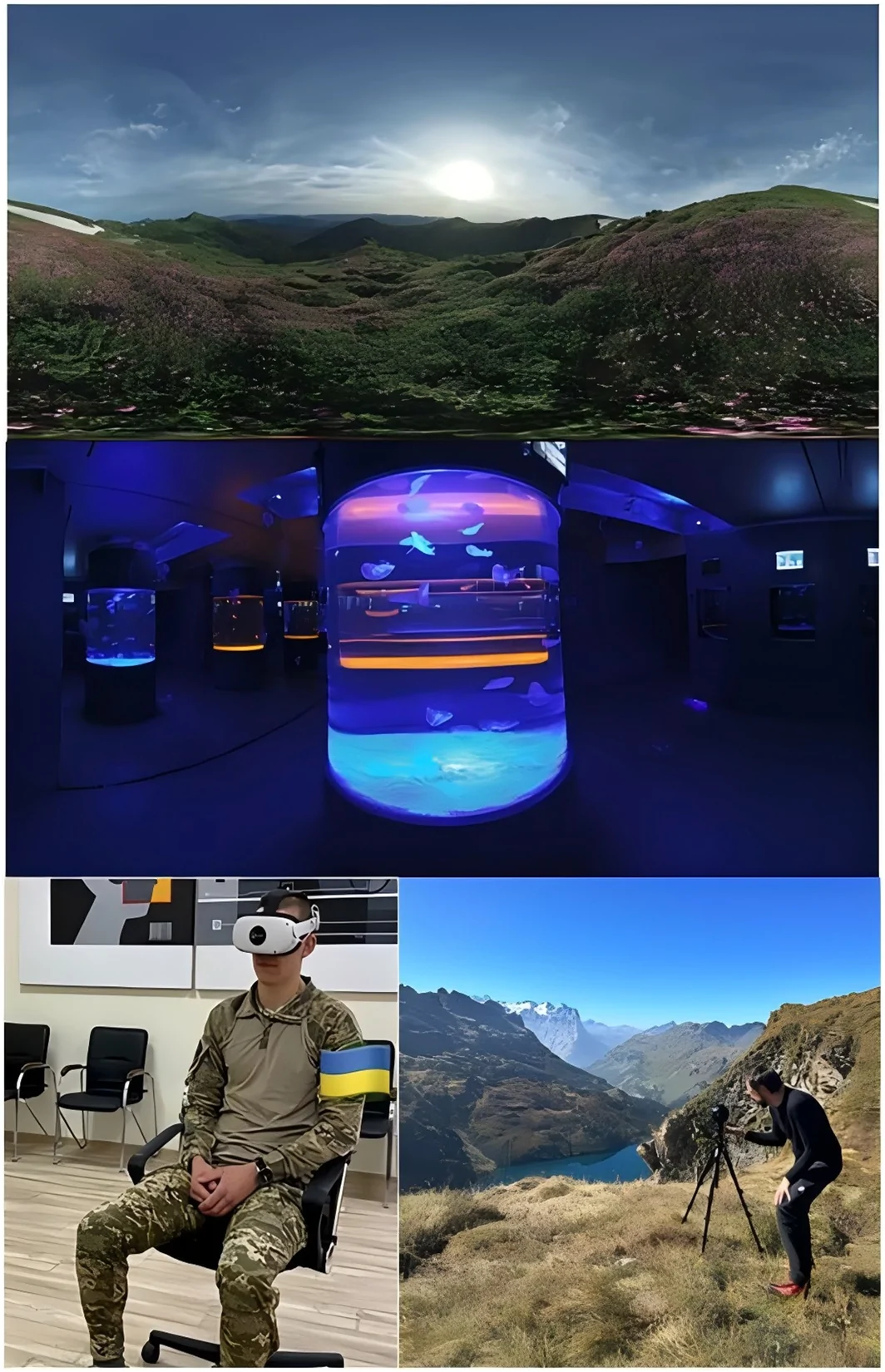
VR treatment using a Meta Quest 2 head‐mounted device^27^. VR, virtual reality.

However, current VR devices have several limitations, as detailed in Supporting Information S1: Table S2. To address these limitations, future optimisation should adopt a three‐pronged approach—cold‐resistant hardware packaging, low‐power programmes adapted to low temperatures and thermostatically controlled storage accessories—to extend battery life and ensure reliable operation under extreme cold conditions. Additionally, incorporating metal shielding around wired radiofrequency circuits may reduce electromagnetic interference between internal and external signals. With continued technological improvements, VR devices may become suitable for reliable deployment in extreme environments.

### Diaphragmatic breathing training

Diaphragmatic breathing training is an evidence‐based intervention for stress and anxiety and may also be beneficial in PTSD treatment. However, in Arctic environments characterised by extreme cold and a high risk of radiation exposure, military personnel may experience increased physiological stress and psychological anxiety. Mild traumatic brain injury (mTBI) may also co‐occur as a result of operational duties in these environments. Without real‐time guidance and supervision, conventional diaphragmatic breathing training can be difficult to learn and may be associated with poor adherence. Technology‐assisted delivery may therefore help improve the effectiveness of the intervention. Wallace et al.^28^ developed BreatheWell Wear, a mobile intervention integrating a smartwatch with a smartphone application, as shown in figure 2. The system supports diaphragmatic breathing training through reminders, biofeedback and visual, tactile and auditory guidance. In a prospective pilot clinical trial involving participants with mTBI and PTSD, the intervention improved attainment of personal goals, treatment adherence and mastery of diaphragmatic breathing techniques. It also reduced the difficulty of remembering and performing the exercises and was associated with lower stress levels. Clinically meaningful improvements were observed in symptoms of PTSD, anxiety and depression, supporting the feasibility of mobile technology‐assisted diaphragmatic breathing training for trauma‐related psychological disorders.

**FIGURE 2:**
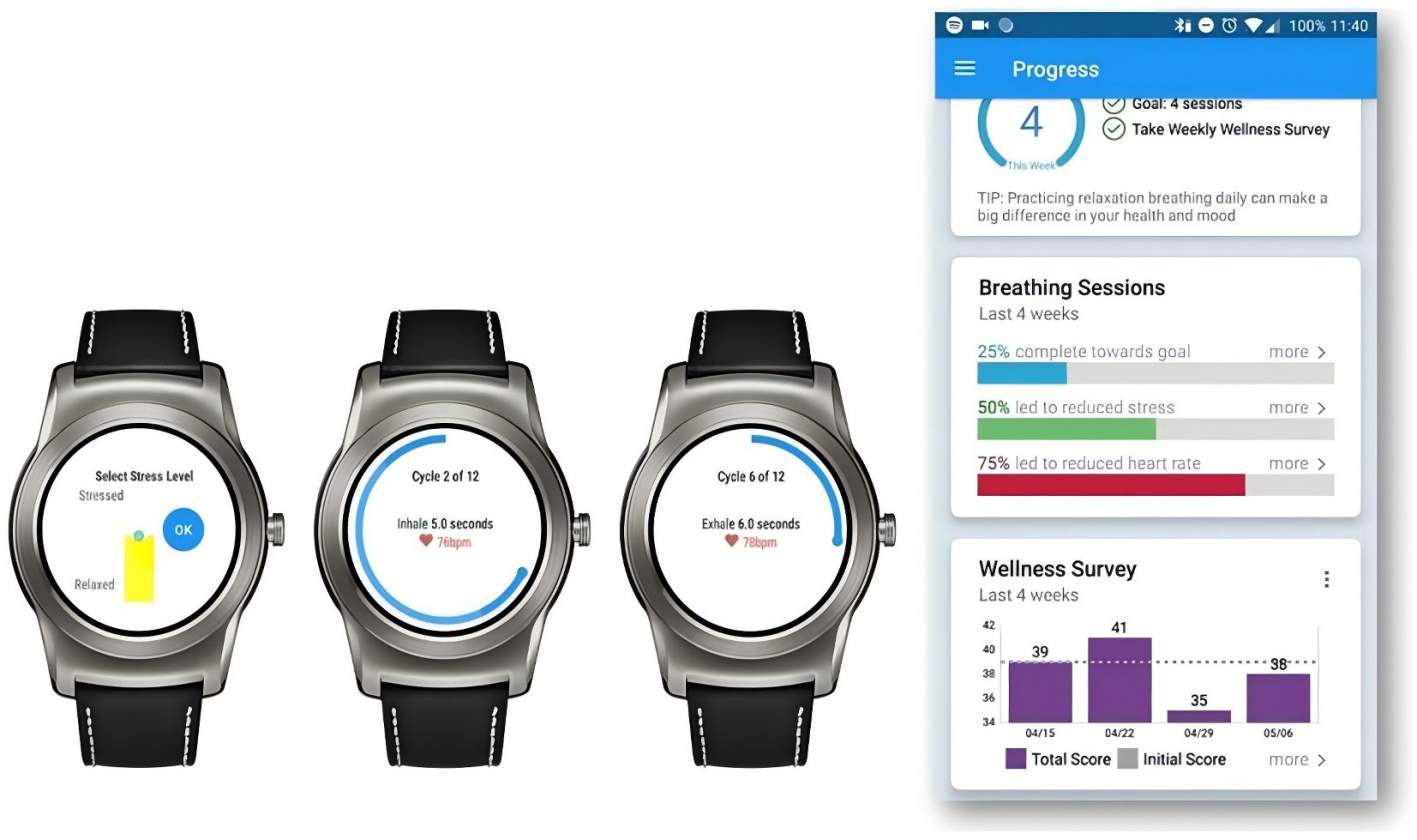
BreatheWell Wear^28^.

Military personnel deployed in cold regions or environments involving a risk of radiation exposure may experience prolonged physiological stress, whereas environmental stimuli may disrupt their breathing patterns. Diaphragmatic breathing training may help regulate autonomic function and alleviate physical tension and anxiety caused by environmental stress, thereby addressing some of the physiological and psychological needs of personnel with PTSD in these settings.^29^ Breathing training tools based on mobile technology can overcome some of the barriers to on‐site intervention by providing portable, real‐time guidance to frontline personnel when psychological services and regular face‐to‐face support are difficult to access.^30^ However, like VR devices, this technology has limitations in extreme cold and radiological environments and therefore requires further optimisation to improve its stability.

## CONCLUSIONS AND FUTURE DIRECTIONS

Cold environments involving a risk of radiation exposure create combined cold‐ and radiation‐related stress, which may contribute to distinctive and complex mechanisms underlying PTSD in military personnel. Current research has preliminarily elucidated the pathogenic pathways involving neuroendocrine mechanisms and the brain–gut–microbiota axis and has identified three types of interventions with clinical potential: precision individualised treatment informed by neuroimaging and machine learning, psychological resilience training and technology‐assisted interventions such as immersive VR therapy and wearable device‐guided diaphragmatic breathing training. However, three major gaps remain: the lack of large military cohorts, the technical limitations of intervention devices used in extreme environments and the limited clinical validation of interventions targeting the gut microbiota. We therefore propose three priority research directions for future investigation:

- Establish a large prospective cohort of military personnel involved in radiological operations in China's cold regions, integrating physiological biomarkers, gut microbial profiles, neuroimaging measures and psychological assessments to develop a multidimensional early‐warning system for PTSD risk stratification.
- Optimise VR and wearable breathing‐intervention devices by incorporating cold‐resistant, radiation‐resistant and low‐power components and evaluate their long‐term effectiveness for PTSD in randomised controlled trials.
- Elucidate the mechanisms of the brain–gut–microbiota axis under combined cold‐ and radiation‐related stress and develop interventions targeting the gut microbiota to alleviate PTSD symptoms in military personnel.

Future research should integrate multiomics, neuroimaging and data from mobile and wearable devices to enable precision monitoring and context‐adapted interventions for PTSD associated with radiological operations in cold regions. Such research may help protect the mental health and sustained operational capacity of military personnel.

## AUTHOR CONTRIBUTIONS

**Hui Wang:** writing – original draft. **Yongxian Zhang:** writing – original draft. **Chuanyu Zhu:** writing – original draft. **Dawei Zhang:** writing – original draft. **Peng Zhou:** writing – original draft. **Chenglin Sang:** writing – review and editing. All authors approved the final manuscript.

## FUNDING INFORMATION

The study was supported by the Project of Shandong First Medical University Affiliated Provincial Hospital (2024053DCS003).

## CONFLICT OF INTEREST STATEMENT

The authors declare no conflicts of interest.

## CONSENT

Not applicable.

## ETHICS STATEMENT

Not applicable.

## Supporting information

Supporting Information S1

Figure S1
