# Supplementary material for: Research progress on intervention strategies for post‐traumatic stress in military personnel exposed to cold environments and radiological threats
Source: Gen Psychiatr. 2026 Oct 29;39(6):e70061. doi: 10.1002/gps3.70061 (PMC13717602; doi:10.1002/gps3.70061)
Supplement: Supplementary file 1 [file GPS3-39-e70061-s001.docx]

Supplemental material

We retrieved relevant literature from the PubMed and Web of Science databases using a predefined search strategy. The complete search formula is presented in figure 1. We acknowledge that confining the search scope to only two databases may result in the omission of some relevant literature. However, since this is a narrative literature review rather than a systematic review, our objective is to summarise the latest research findings and provide reference value for future research rather than conducting a detailed multi-database search.

**Figure 1** The flowchart of the research screening process. The search was conducted using PubMed and Web of Science, with a predefined search strategy, and the screening conditions were set as human studies and English-language literature, covering the years from database establishment to 2026. The initial search yielded a total of 173 articles (147 from PubMed and 26 from Web of Science). After removing duplicates, 173 articles remained. After a preliminary screening of titles and abstracts, 94 articles were excluded, leaving 79 for full-text evaluation. Among them, 26 were excluded due to not meeting the inclusion criteria, and another 53 underwent full-text evaluation. After further review, 23 were excluded, resulting in a total of 30 studies being included in this review

| **Table S1 Aggravating factors of PTSD in cold region and nuclear environment** | | | |
| --- | --- | --- | --- |
| Aggravating factors |  | Risk factors and examples | Mechanisms of influence |
| Physiological factors | Baseline physiological traits and genetic susceptibility | Age, sex, genetic polymorphisms (stress-sensitive genes such as 5-HTTLPR and FKBP5), and underlying physical conditions (gastrointestinal diseases and immune dysfunction). | These factors determine an individual's baseline physiological threshold for stress, while genes and underlying physical conditions directly affect the stress-regulatory capacity of the neuroendocrine system, thereby amplifying the physiological and psychological responses following trauma. |
|  | Physiological stress and injury in cold regions | Long-term cold exposure, hypothermia/frostbite; cold-induced sleep deprivation and chronic fatigue accumulation; and gastrointestinal dysfunction, respiratory diseases, and other somatic discomforts triggered by cold environment conditions. | Persistent cold stress disrupts neuroendocrine and immune functions. Somatic discomfort continuously reinforces the individual's negative perception, weakens psychological coping capacity, and produces an additive effect with traumatic psychology. |
|  | Physiological damage related to nuclear radiation and stress | Cellular damage and inflammatory response induced by nuclear radiation exposure, as well as radiation-triggered somatic symptoms (nausea, fatigue, damage to the skin and mucous membranes, and haematopoietic dysfunction). | Radiation directly damages the brain's emotion regulation centres, including the hippocampus and amygdala. Somatic symptoms and long-term health risks continuously activate the individual's sense of fear and loss of control, thereby consolidating traumatic memory. |
| Psychological factors | Trauma-related cognitive biases | Excessive fear of nuclear radiation, catastrophising cognition; negative expectations and perceived uncontrollability of cold-region environments, self-attribution of traumatic events; excessive self-blame. | Cognitive biases persistently amplify the negative effects of traumatic events, solidify an individual's sense of fear and hopelessness, and impede the normal processing and extinction of traumatic memories, thereby serving as a core psychological driver of PTSD exacerbation. |
|  | Maladaptive coping styles and emotion regulation ability | Avoidant coping; negative attribution; rumination. | Avoidant coping impedes the processing of traumatic memories, while rumination persistently reinforces negative emotions, ultimately resulting in the failure of traumatic memories to undergo normal extinction. |
| Environmental factors | | Harsh natural conditions in cold regions, including extreme low temperatures, snowstorms, polar nights, and high altitudes; survival environments characterized by nuclear radiation contamination, environmental degradation, and resource scarcity; enclosed and isolated field deployment environments, cut off from the outside world. | Extreme physical environments continuously impose dual physiological and psychological pressures on individuals, reinforce the core perception of "survival threat," maintain individuals in a prolonged state of stress arousal, and significantly increase the risk of PTSD exacerbation. |
| Operational factors | | High-intensity sustained operations, long-term field patrols and operations in nuclear-contaminated areas; high-risk missions involving direct contact with nuclear radiation and emergency response in low-temperature environments, tasks that are highly demanding, with low tolerance for error, and subject to strict disciplinary and accountability constraints. | High-risk, high-intensity operations expose individuals to dual stressors over prolonged periods, maintaining them in a sustained high-pressure state, significantly increasing the probability of traumatic events, while simultaneously undermining their psychological coping capacity. |

| **Table S2 Intervention and treatment strategies for PTSD in cold region nuclear environments** | | | | |
| --- | --- | --- | --- | --- |
| **Intervention classification** | | **Specific intervention strategy** | **Environmental adaptation advantage** | **Extant limitations** |
| **Individualised medicine** | | **Based on neuroimaging (fMRI/EEG), biomarkers, genetic testing, and machine learning-driven predictive models utilising big data** | 1. **Objective quantifiable measures minimise subjective bias inherent in manual assessment.** 2. **It is applicable to large-scale stratified screening of military personnel undergoing prolonged field training.** | 1. **The cost of the detection equipment is high and it is difficult to deploy it quickly in extremely special environments.** 2. **Multi-dimensional sample collection procedures are complex and pose significant challenges for field implementation.** 3. **The limited training dataset specific to this population leads to poor model generalisability.** |
| **Strategies for enhancing psychological resilience** | | **A comprehensive intervention framework centred on GAT-based multi-dimensional assessment, personalised self-improvement plans, and grassroots psychosocial resilience key personnel training.** | 1. **It simultaneously addresses the combined physical and psychological impairments induced by low temperatures and prolonged nuclear radiation in cold regions.** 2. **It eliminates the need for large-scale medical equipment, making it suitable for widespread deployment at remote field training sites.** | **Owing to the protracted training cycle, achieving full implementation across all personnel is challenging under high-intensity operational duties.** |
| **New therapy** | **Applications of virtual reality technology** | **Exposure therapy is administered through head-mounted devices that deliver 360-degree immersive scenes.** | 1. **It eliminates the need to enter actual hazardous zones, thereby ensuring the safety of intervention personnel.** 2. **The device is portable and the intervention protocol is standardised, serving a dual purpose of psychological rehabilitation and hands-on military medical simulation training** | 1. **.Battery life of smart devices is significantly reduced under low-temperature environments.** 2. **Electronic devices are at risk of signal interference and malfunction under high-radiation environments.** |
|  | **Respiratory training** | **BreatheWell Wear smartwatch and mobile app-based multimodal biofeedback breathing training.** | 1. **The device is compact and portable, enabling training to be conducted around the clock during field duties and intervals between nuclear operations.** 2. **It provides real-time biofeedback that adapts to fluctuating somatic stress states of service personnel under cold-region environmental conditions.** |  |
